# Multidimensional Assessment of Athletic and Non-Athletic Female Students Through Analysis of BMI, Body Perception, Objectification, and Attitudes Towards the Ideal Body

**DOI:** 10.3390/bs15111454

**Published:** 2025-10-25

**Authors:** Dana Badau, Adela Badau, Dragos Florin Teodor, Corina Claudia Dinciu, Victor Dulceata, Dan Cristian Mănescu, Catalin Octavian Mănescu, Marin Florin Litoi, Alina-Mihaela Stoica

**Affiliations:** 1 Department of Physical Education and Special Motricity, Transylvania University of Brasov, 500036 Brasov, Romania; 2 Department of Motor Performance, Transylvania University of Brasov, 500036 Brasov, Romania; 3 Faculty of Physical Education and Sports, Ovidius University of Constanta, 900470 Constanta, Romania; 4 Department of Physical Education and Sport, Academy of Economy Sciences Bucharest, 010374 Bucharest, Romania; 5 Department of Physical Education and Sports, University of Bucharest, 050107 Bucharest, Romania

**Keywords:** body image perception, objectified body consciousness, body size, body dissatisfaction, body stereotypes

## Abstract

This study critically examines the multidimensional differences in body image perceptions among female students who participate in regular sports activities compared to their sedentary counterparts. The investigation involved a sample of 436 female students divided into two distinct groups: the sports group (GS, n = 180), consisting of participants from physical education and sports disciplines, and the non-sports group (GNS, n = 256). Anthropometric measurements such as height, weight, and body mass index (BMI) were systematically taken, along with the administration of three validated psychometric tools: the Silhouette Rating Scale (SRS) to assess body perception and satisfaction, the Objectified Body Consciousness Scale (OBC) to evaluate body objectification, and the Ideal Body Stereotype Scale-Revised (IBIS-R) to analyze perceptions of ideal body stereotypes. Notably, body dissatisfaction (SRS-D) showed the strongest correlation with BMI in both groups, with the non-athletic group displaying slightly higher correlation coefficients (r = 0.940) compared to the athletic group (r = 0.904; *p* < 0.001). Additionally, stereotypes related to the ideal body (IBIS-R) were strongly correlated with BMI in the non-athletic group (r = 0.846), whereas the athletic group showed a slightly lower correlation (r = 0.805). The body objectification measure (OBC) demonstrated moderate correlations, with the non-athletic group showing stronger associations (r = 0.394 vs. r = 0.352). Linear regression analysis revealed that non-athletic individuals exhibited higher predictive validity, characterized by greater R^2^ values and stronger correlations between physical and psychosocial factors. The results indicate that participation in sports serves as a protective factor against negative body image, shown by weaker correlations in the sports group. This research suggests that engaging in physical activities is associated with healthier body profiles and a more positive body image, leading to greater satisfaction and more realistic perceptions of body size.

## 1. Introduction

Multidimensional assessments that simultaneously examine body perception, objectification, and ideal body stereotypes concerning objective anthropometric indices are rare in the literature. Previous studies have explored the relationship between physical activity and body image, emphasizing positive correlations between sports participation and body satisfaction (Jankauskiene et al., 2023; Cox et al., 2017). The literature suggests that engaging in sports activities can serve as a protective and motivating factor against developing a negative body image by promoting a functional view of the body, focusing on abilities and performance (Baceviciene et al., 2023; Goicochea et al., 2022; Balciuniene et al., 2022). Recent epidemiological data indicate that 40–60% of young women experience moderate to severe body dissatisfaction (Vandenbosch et al., 2022; Jiotsa et al., 2021), and the prevalence of eating disorders and sedentary behavior among university students has increased by 30% over the past decade (Herreros-Irarrázabal et al., 2024; Müller et al., 2022).

Body image is a complex psychological concept that encompasses a person’s perceptions, thoughts, feelings, and actions regarding their own body. It is a key predictor of mental health and psychological well-being (Han et al., 2023; Miranda et al., 2021). Body objectification theory suggests that women are socialized to view their bodies from an outsider’s perspective, which can lead to self-monitoring, body shame, and feeling less in control of their bodies (Ma et al., 2025; Dimas et al., 2021; Ainley & Tsakiris, 2013). Media, family, and peers are significant influences that encourage the internalization of the thin ideal, which can lead to increased body dissatisfaction (Aparicio-Martinez et al., 2019; Fitzsimmons-Craft et al., 2014).

Positive body image perception is an essential determinant of psychological functioning and the development of healthy behaviors (Gualdi-Russo et al., 2022a; Linardon et al., 2022). Studies show strong inverse correlations between positive body image and depression, anxiety, or eating disorders, with body dissatisfaction being a strong predictor of these conditions (Torres et al., 2024; O’Dea & Abraham, 2000). The process of objectification directs cognitive resources towards compulsive body monitoring, affecting psychological adaptation (Himmerich & Mirzaei, 2024; Gualdi-Russo et al., 2022b). A positive body image supports global self-esteem, identity coherence, and personal autonomy (Crerand et al., 2024; Lewis-Smith et al., 2019). Acceptance of one’s own body decreases dependence on external validation and vulnerability to social pressures (Himmerich & Mirzaei, 2024; Azevedo & Azevedo, 2023). At the interpersonal level, body acceptance is associated with relational satisfaction and psychological resilience (Gualdi-Russo et al., 2022b; Aboody et al., 2020). Interventions aimed at improving body image significantly reduce the risk of behavioral disorders, justifying their systematic inclusion in prevention and psychological intervention programs (Crerand et al., 2024; Linardon et al., 2022). Studies have shown that psychological aspects are essential in the positive perception of body image, and physical activity can be a decisive support for improving it and increasing body satisfaction (Marquez et al., 2024; Gualdi-Russo et al., 2022b).

The methodological and conceptual gaps identified from the systematic review of the literature on body image in both sports and non-sports populations are numerous. Several studies approach body image in a fragmented and unidimensional manner, focusing on a single component (usually body satisfaction) at the expense of a multidimensional assessment that includes perception, objectification, and body stereotypes (Vendemia, 2024; Kellie et al., 2019). This limitation hinders a comprehensive understanding of how sports activity affects various aspects of body image. Another significant gap is the lack of integration between objective and subjective measurements in existing research. Studies assessing body image rarely include objective anthropometric measures (BMI) to externally validate self-reported body perceptions, which limits the ability to identify discrepancies between physical reality and subjective perception—an essential factor in understanding body image distortions (Tort-Nasarre et al., 2021; Goonapienuwala et al., 2019). Discrepancies between self-reported and objectively measured BMI were quantified using difference scores (objective BMI and self-reported BMI) and classification accuracy across BMI categories. Although objectification theory significantly advances our understanding of female body image, its application within the context of sports remains underexplored, with a lack of systematic assessments on how sports influence processes such as self-monitoring and body shame (Dimas et al., 2021; Thøgersen-Ntoumani et al., 2011). Several studies have relied on simple correlational designs without analyzing inter-scale relationships or predictive factors, which restricts the understanding of complex relationships between body image variables and the identification of key intervention targets (Wallner et al., 2022; Choukas-Bradley et al., 2022).

These gaps underscore the need for a comprehensive study that simultaneously assesses body perception, objectification, and ideal body stereotypes in relation to objective anthropometric measurements, utilizing validated psychometric tools in both athletic and non-athletic groups. The current study adopts this integrated approach by thoroughly assessing body image through four key components: objective anthropometric measurements (BMI) and three validated psychometric instruments—Silhouette Rating Scale for body perception (Lombardo et al., 2022; Hao et al., 2022), Objectified Body Consciousness Scale for body objectification (Söyünmez et al., 2024; Forbes et al., 2006), and Ideal Body Stereotype Scale-Revised for ideal body stereotypes (Sicilia et al., 2022; Thompson et al., 2018). Its theoretical originality lies in applying both body objectification theory and the body ideal stereotypes model simultaneously within the specific context of athletic versus non-athletic female students. This complex approach offers a new perspective on how sports activity can mitigate the negative impacts of sociocultural pressures on female body image. The innovative aspect of the analysis is the use of a correlational model that examines not only group differences but also complex relationships among variables, unique correlations, and factors associated with a positive body image. The study’s contribution to objectification theory is noteworthy, as it explores how sports activity influences body self-perception, body shame, and body control (Dias et al., 2021; Stewar et al., 2003). By measuring how much athletes and non-athletes internalize societal ideals, the research helps identify body shape types. Focusing on modifiable protective factors, such as sports activity, the study aims to inform evidence-based intervention programs that can prevent body image issues and motivate proactive behaviors.

This study aims to evaluate the differences in body image between female students who systematically practice sports and those who are sedentary by multidimensionality, analyzing the relationship between objective body mass index (BMI) and subjective body image indices.

The study hypothesis suggests that female sports students will have a significantly more positive perception of body image across all evaluated dimensions (normoweight category of BMI, accurate body perception, reduced objectification, and low internalization of stereotypes about the ideal body) compared to non-sports students, confirming the protective role of regular sports activity against the development of a negative body image.

## 2. Materials and Methods

### 2.1. Study Design

The research was conducted from April to June 2025, involving a comprehensive anthropometric assessment measuring participants’ height, weight, and BMI. Data collection was carried out through online questionnaires using Google Forms, ensuring convenience and accessibility. Both the assessment and questionnaire completion were voluntary, in accordance with ethical standards. The selection of questionnaires was based on recommendations from sports psychology experts and a thorough review of relevant literature. Previous studies support a multidimensional approach to body image, validating the three questionnaires used in this study. The methodology adheres to established guidelines, allowing for a detailed analysis of the link between physical measurements and body image perceptions. Participation was voluntary and required informed consent from each subject of the study prior to data collection. The study complied with the principles of the Declaration of Helsinki and received approval from the Ethics Committee of Transilvania University of Brasov, Faculty of Physical Education and Mountain Sport, under no. 247b/01.10.2024.

### 2.2. Participants

The study involved a total of 436 female students, who were divided into two distinct groups based on their academic specialization. The sports group (SG) included 180 students from physical education and sports, while the non-sports group (NSG) consisted of 256 students from various non-sport disciplines, specifically economics, law, and educational sciences. In the sports group (SG), the participants were mostly female, with an average age of 20.42 years (±1.11). Anthropometric measurements showed an average height of 169.34 cm (±2.37) and an average weight of 60.87 kg (±6.48). Conversely, the non-sports group (NSG) had an average age of 20.41 years (±1.10), with a mean height of 165.28 cm (±2.34) and an average weight of 66.02 kg (±7.84). The inclusion criteria for the sports group required participants to be actively involved in sports (minimum 3 training sessions of 90 min per week, moderate intensity of effort), maintain good health, and fully complete the provided questionnaires. For the non-sports group, the criteria specified that students should not participate in competition, maintain a semi-sedentary or sedentary lifestyle, and have good health and complete questionnaire submissions. Specifically, participants in the non-athletic group were defined as not involved in physical activity at all or engaged in less than 2 h of structured physical activity per week (with reduced intensity and volume of effort), without regularly participating in organized sports or fitness programs.

### 2.3. Assessment Tools

In the study, we employed anthropometric measurements and three standardized psychometric questionnaires for body image assessment. The anthropometric measurements included height, weight, and BMI calculation according to WHO standards. Validated psychometric instruments used were the Silhouette Rating Scale (SRS) for body perception and satisfaction, the Objectified Body Consciousness Scale (OBC) for body objectification, and the Ideal Body Stereotype Scale-Revised (IBIS-R) for stereotypes about the ideal body.

Questionnaires evaluated in the study:

1. The Silhouette Rating Scale (SRS) is an imaging tool used to assess body image, consisting of a series of nine silhouettes representing either females or males. It was developed to evaluate perceptions of current and ideal body sizes, as well as body dissatisfaction (Lombardo et al., 2022; Hao et al., 2022). The application of this questionnaire involves the following stages:
  - Presentation of the scale: the participant is shown a series of nine silhouettes matching their gender;
  - First assessment—Current body size identification (SRS-C), with the instructions: “Please select the silhouette that best represents your current body size.” The participant chooses a number from 1 (very thin) to 9 (very obese);
  - Second assessment—Ideal body size identification (SRS-I): with the instructions: “Please select the silhouette that represents the body size you would like to have.” The participant again chooses a number from 1 to 9;
  - Calculating body dissatisfaction (SRS-D): the difference is calculated by subtracting SRS-I from SRS-C, representing the measure of body dissatisfaction.
2. The Objectified Body Consciousness Scale (OBC) is a 24-item measure assessing how females perceive their bodies as objects that can be altered after internalizing societal standards. The scale includes three subscales: (1) body surveillance (seeing the body as an outside observer), (2) body shame (experiencing shame when the body does not meet expectations), and (3) appearance body control beliefs (Söyünmez et al., 2024; Forbes et al., 2006; McKinley & Hyde, 1996). Response options: 7-point Likert scale: strongly disagree—1 and strongly agree—7. The sum of each subscale is calculated, as well as the total instrument score; higher scores indicate a greater endorsement of each of the constructs.
3. The Ideal Body Stereotype Scale-Revised (IBISR-6) (n.d.) is a tool used to assess individuals’ perceptions and attitudes towards the ideal body image. It examines factors such as attractiveness, well-being, and societal expectations regarding body image (Physical Attraction, Social Success, Personal Success, Positive Stereotype, and Negative Stereotype). The scale includes six items, each with a 5-point Likert response from ‘strongly disagree’ to ‘strongly agree’ (Sicilia et al., 2022; Thompson et al., 2018).

### 2.4. Statistical Analysis

The study results were analyzed statistically using SPSS 2026 software, a robust tool for data analysis in social sciences. To clarify the relationships among the results, several statistical parameters were used, including the mean (X) to determine the central tendency of the data, standard deviation (SD) to measure dispersion, as well as Skewness and Kurtosis to assess the shape of the data distribution; for data normality we calculate the Shapiro–Wilk, To identify differences between groups, we calculated Student’s *t*-test including Levene test and confidence coefficient (95%CI) with the two lower limits (LL) and upper (UL). Cronbach’s Alpha was calculated, with a minimum threshold of 0.70 to confirm the scales’ dependability. Statistical significance was set at *p* < 0.05, ensuring robust conclusions from the data. Moreover, Pearson correlation coefficients were calculated to examine the strength and direction of relationships between variables. Finally, multiple regression linear analysis was conducted to identify and measure the predictors affecting the outcome variables, providing a deeper understanding of the factors involved in the study. Standardized beta coefficients (β) quantify the size and direction of the association between predictors and dependent variables, controlling for the effects of other predictors in the model. The magnitude of the association is interpreted according to the following criteria: values close to 0 indicate weak associations, β = 0.1–0.3 signify weak to moderate associations, β = 0.3–0.5 reflect moderate associations, β > 0.5 denote strong associations, and β > 0.8 indicate very strong associations between the variables analyzed.

## 3. Results

In Table 1, the data on the distribution of subjects and their percentage values according to BMI scores and the specific subscales of the three standardized psychometric questionnaires are shown.

The data analysis in Table 1 shows notable differences between the sports group (GS, n = 180) and the non-sports group (GNS, n = 256) regarding body composition and perceptions of body image. Body mass index (BMI) is more favorably distributed in the sports group, with 83.9% of participants in the normoponderal range (18.5–24.99 kg/m^2^), compared to 58.2% in the non-sports group. The non-sports group (GNS) has a higher prevalence of overweight (41.4% vs. 16.1%) and one case of grade 1 obesity (0.4%). Body size perception (SRS-C) shows that the sports group (GS) has a higher proportion of individuals perceiving themselves as thin (23.3% vs. 10.9%), whereas the non-sports group (GNS) tends toward perceiving a larger body size (28.5% vs. 10.6%). Body satisfaction (SRS-D) differs significantly; the sports group (GS) reports 31.7% with perfect satisfaction and 51.1% with mild dissatisfaction, while the non-sports group (GNS) has no cases of perfect satisfaction and mostly moderate dissatisfaction (96.9%). Body objectification (OBC) levels are low in both groups (98.9% vs. 96.9%), with slight differences in distribution. Ideal Body Stereotypes (IBIS-R) show that the athletic group scores mostly in the low-moderate (31.7%) and moderate (52.8%) categories, while the non-athletic group has a more even split, with 40.6% in the moderate-high category. These findings suggest that engaging in athletic activity correlates with a more favorable anthropometric profile and a more positive body image, characterized by greater satisfaction and more realistic perceptions of body size.

In Table 2, Table 3, Table 4, Table 5, Table 6, Table 7 and Table 8, descriptive statistics, correlation analyses, and linear regression results for the study variables are presented.

Descriptive statistics reveal systematic differences between the sports group (GS) and the non-sports group (GNS) for the investigated variables. Body mass index presents significantly different mean values (M = 22.285, SD = 2.251 for GS; M = 24.159, SD = 2.716 for GNS), with positive asymmetric distributions in both groups (skewness = 0.513 GS; 0.312 GNS). Body image dimensions reveal distinctive patterns: perceived current body size (SRS-C) is lower in GS (M = 4.538, SD = 1.330) compared to GNS (M = 5.543, SD = 1.483), while body dissatisfaction (SRS-D) is substantially lower in GS (M = 1.222, SD = 1.193) compared to GNS (M = 2.144, SD = 1.337). Objectified Body Consciousness (OBC) scales demonstrate that GNS score higher on body surveillance (M = 4.335, SD = 1.019) and body shame (M = 3.621, SD = 1.204) compared to GS (M = 3.555, SD = 0.898; M = 2.650, SD = 1.070, respectively), while body appearance control is more pronounced in GS (M = 5.216, SD = 1.295) versus GNS (M = 4.027, SD = 1.448). Ideal body stereotypes (IBIS-R) reflect consistently higher mean values in the GNS for all subdimensions: physical attractiveness (M = 3.550 vs. 3.222), social success (M = 3.039 vs. 2.433), personal success (M = 3.261 vs. 2.838), and negative stereotypes (M = 2.722 vs. 2.272). The skewness and flattening coefficients indicate close to normal distributions for most variables, confirmed by the Shapiro–Wilk test (*p* > 0.05). The internal consistency of the instruments is adequate, with Cronbach’s alpha coefficients ranging between 0.769 and 0.968, validating the reliability of the measurements performed.

**Table 3:** Student’s *t*-test analysis of the study.

Parameters	Levene’s Test for Equality of Variances		*t*-Test				
	F	*p*	t	*p*	Mean Difference	95% CI Lower	95% CI Upper
BMI (kg/m^2^)	16.153	0.000	−7.599	0.000	−1.873	−2.358	−1.389
Current Size (SRS-C)	5.521	0.019	−7.256	0.000	−1.004	0.138	−1.276
Ideal Size (SRS-I)	0.139	0.009	−1.409	0.016	−0.077	−0.186	0.030
Body Dissatisfaction (SRS-D)	5.825	0.016	−7.437	0.000	−0.926	−1.171	−0.681
Body surveillance	12.497	0.000	−8.259	0.000	−0.780	−0.966	−0.594
Body shame	5.933	0.015	−8.672	0.000	−0.971	−1.191	−0.751
Appearance body control	8.422	0.004	8.813	0.000	1.189	0.924	1.454
Score Total OBC	7.842	0.005	−1.383	0.017	−0.020	−0.048	0.008
Physical Attraction	47.586	0.000	−7.062	0.000	−0.389	−0.498	−0.281
Social Success	2.774	0.006	−8.018	0.000	−0.605	−0.754	−0.457
Personal Success	3.542	0.001	−6.344	0.000	−0.422	−0.553	−0.291
Positive Stereotype	3.542	0.041	−6.344	0.000	−0.422	−0.553	−0.291
Negative Stereotype	8.945	0.003	−8.561	0.000	−0.450	−0.553	−0.347
Score Total IBIS-R	3.542	0.031	−6.344	0.000	−0.422	−0.553	−0.291

F—F test value, *p*—level of probability, t—value of Student’s *t*-test.

Student’s *t*-test analysis revealed significant differences between groups for all variables analyzed. The Levene test indicates unequal variances (*p* < 0.05) for BMI, current size perception, body dissatisfaction, body surveillance and shame, appearance control, total OBC score, physical attractiveness, social success, personal success, positive stereotypes, negative stereotypes, and total IBIS-R score, while ideal size presents homogeneous variances (*p* = 0.009 > 0.05). For BMI, a mean difference of −1.87 kg/m^2^ is observed (t = −7.60, *p* < 0.001, 95% CI: −2.36 to −1.39), indicating that athletes present substantially lower values compared to non-athletes. The perception of current size is 1.00 points lower (t = −7.26, *p* < 0.001, 95% CI: 0.14 to −1.28), and ideal size differs significantly by −0.08 points (t = −1.41, *p* = 0.016, 95% CI: −0.19 to 0.03). Body dissatisfaction shows a difference of −0.93 points (t = −7.44, *p* < 0.001, 95% CI: −1.17 to −0.68), body surveillance of −0.78 points (t = −8.26, *p* < 0.001, 95% CI: −0.97 to −0.59), and body shame of −0.97 points (t = −8.67, *p* < 0.001, 95% CI: −1.19 to −0.75). Body control is the only exception with a positive difference of +1.19 points (t = 8.81, *p* < 0.001, 95% CI: 0.92 to 1.45), suggesting higher values in athletes. The total OBC score shows a small but significant difference of −0.02 points (t = −1.38, *p* = 0.017, 95% CI: −0.05 to 0.01). The stereotype dimensions all show significant negative differences: physical attraction −0.39 (t = −7.06, *p* < 0.001, 95% CI: −0.50 to −0.28), social success −0.61 (t = −8.02, *p* < 0.001, 95% CI: −0.75 to −0.46), personal success −0.42 (t = −6.34, *p* < 0.001, 95% CI: −0.55 to −0.29), positive stereotypes −0.42 (t = −6.34, *p* < 0.001, 95% CI: −0.55 to −0.29), negative stereotypes −0.45 (t = −8.56, *p* < 0.001, 95% CI: −0.55 to −0.35) and IBIS-R total score −0.42 (t = −6.34, *p* < 0.001, 95% CI: −0.55 to −0.29). These results reflect the positive impact of regular physical activity on body image, with athletes benefiting from a lower BMI, less body dissatisfaction and shame, and reduced internalization of weight-related stereotypes, probably due to the positive feedback generated by physical performance and body functionality. The group of athletes demonstrates greater control over their physical appearance, which may reflect the active body monitoring required in sports practice.

The correlation analysis presented in the table reveals strong and statistically significant relationships between body mass index (BMI) and the components of the SRS (Silhouette Rating Scale) in both study groups (Table 4). The correlations with BMI show very strong and positive associations with most variables. In the sports group (GS), BMI has a strong correlation with SRS-C (r = 0.937, *p* < 0.001), SRS-I (r = 0.283, *p* < 0.001), and SRS-D (r = 0.904, *p* < 0.001). The non-sports group (GNS) shows similar correlations, but slightly stronger: SRS-C (r = 0.957, *p* < 0.001), SRS-I (r = 0.296, *p* < 0.001), and SRS-D (r = 0.940, *p* < 0.001). The relationships between the SRS components indicate different patterns between the groups. SRS-C correlates strongly with SRS-D in both groups (GS: r = 0.895; GNS: r = 0.929, *p* < 0.001), suggesting that the perception of current body size is closely linked to the level of body dissatisfaction. The correlation of SRS-C with SRS-I is moderate in both groups (GS: r = 0.441; GNS: r = 0.438, *p* < 0.001). The relationship between SRS-I and SRS-D shows a noticeable difference between groups: while the non-athletic group exhibits a weak but significant correlation (r = 0.075, *p* = 0.234), the athletic group does not show a significant correlation (r = −0.005, *p* = 0.944), indicating that among athletes, ideal body image does not influence body dissatisfaction. These results suggest that in both groups, there is a strong agreement between objective measurements (BMI) and subjective perceptions of body size, with subtle differences in how body ideals affect body satisfaction between athletic and non-athletic populations.

**Table 4:** Pearson correlations between BMI and Silhouette Rating Scale (SRS) for GS (the upper right diagonal of the table) and GNS (italic letters—the lower left diagonal of the table).

Variables	Correlation	BMI (kg/m^2^)	SRS-C	SRS-I	SRS-D
BMI (kg/m^2^)	r	-	0.937 **	0.283 **	0.904 **
	*p*	-	0.000	0.000	0.000
Current Size (SRS-C)	r	*0.957* **	-	0.441 **	0.895 **
	*p*	*0.000*	-	0.000	0.000
Ideal Size (SRS-I)	r	*0.296* **	*0.438* **	-	−0.005
	*p*	*0.000*	*0.000*	-	0.944
Body Dissatisfaction (SRS-D)	r	*0.940* **	*0.929* **	*0.075*	-
	*p*	*0.000*	*0.000*	*0.234*	-

** Correlation is significant at the 0.01 level (2-tailed). Italics for GNS; normal letters for GS.

In Table 5, the correlation analysis between BMI and the OBC (Objectified Body Consciousness) scale uncovers complex patterns of association that vary notably between the sports and non-sports groups. The correlations with BMI show strong and positive links with the negative components of body objectification in the sports group (GS). BMI is very strongly correlated with Body Surveillance (r = 0.934, *p* < 0.001) and Body Shame (r = 0.919, *p* < 0.001), indicating that higher anthropometric measures are linked to increased self-monitoring and body discomfort. The strong negative correlation with Body Control (r = −0.924, *p* < 0.001) suggests that higher BMI relates to a decreased sense of control over the body. The total OBC score has a moderate correlation with BMI (r = 0.352, *p* < 0.001). The non-athletic group (GNS) shows similar correlations, but with slightly different strengths: Body Surveillance (r = 0.943, *p* < 0.001), Body Shame (r = 0.917, *p* < 0.001), and Body Control (r = −0.917, *p* < 0.001). The total OBC score shows a somewhat higher correlation (r = 0.394, *p* < 0.001). The intercorrelations among the OBC subcomponents indicate that this is a strong factor. Surveillance and Body Shame correlate very strongly and positively in both groups (GS: r = 0.953; GNS: r = 0.921, *p* < 0.001), while both correlate negatively and very strongly with Body Control (r ≈ −0.976, *p* < 0.001), confirming the multidimensional nature of objectification. Total OBC scores have moderate correlations with the subcomponents, ranging from r = 0.283–0.299 for the positive dimensions and r = −0.205 to −0.223 for Body Control, suggesting that the overall score reflects all the measured dimensions in a balanced way. These findings support the idea that body objectification is closely linked to anthropometric traits, with similar patterns in both groups but slightly different magnitudes, possibly reflecting the protective effects of sports activity.

**Table 5:** Correlation Pearson between BMI and Objectified Body Consciousness Scale (OBC) for GS (the upper right diagonal of the table) and GNS (italic letters—the lower left diagonal of the table).

Variables	Correlation	BMI (kg/m^2^)	Body Surveillance	Body Shame	Appearance Body Control	Scor Total OBC
BMI (kg/m^2^)	r	-	0.934 **	0.919 **	−0.924 **	0.352 **
	*p*	-	0.000	0.000	0.000	0.000
Body surveillance	r	0.943 **	-	0.953 **	−0.977 **	0.289 **
	*p*	*0.000*	-	0.000	0.000	0.000
Body shame	r	*0.917* **	*0.921* **	-	−0.976 **	0.283 **
	*p*	*0.000*	*0.000*	-	0.000	0.000
Appearance body control	r	*−0.917* **	*−0.976* **	*−0.951* **	-	−0.223 **
	*p*	*0.000*	*0.000*	*0.000*	-	0.003
Score Total OBC	r	*0.394* **	*0.294* **	*0.299* **	*−0.205* **	-
	*p*	*0.000*	*0.000*	*0.000*	*0.001*	-

** Correlation is significant at the 0.01 level (2-tailed). Italics for GNS; normal letters for GS.

The correlation analysis (Table 6) between BMI and the IBIS-R (Ideal Body Stereotype Scale-Revised) scale shows strong and consistent links between body measurements and ideal body stereotypes in both study groups. The correlations with BMI reveal strong and positive relationships with all dimensions of body stereotypes in the sports group (GS). The strongest links are found with Negative Stereotype (r = 0.906, *p* < 0.001) and Social Success (r = 0.899, *p* < 0.001), followed by Physical Attraction (r = 0.861, *p* < 0.001). Personal Success and Positive Stereotype show similar moderate-strong correlations (r = 0.805, *p* < 0.001), and the overall IBIS-R score correlates at r = 0.805 (*p* < 0.001). The non-athletic group (GNS) displays similar patterns, but with slightly higher correlations for most dimensions: Social Success (r = 0.902, *p* < 0.001), Physical Attraction (r = 0.890, *p* < 0.001), and Negative Stereotype (r = 0.884, *p* < 0.001). Personal Success and Positive Stereotype have identical correlations (r = 0.846, *p* < 0.001), similar to the total IBIS-R score. The intercorrelations among the IBIS-R subcomponents reveal a complex internal structure. Perfect correlations are seen between certain dimensions in the athletic group: Personal Success with Positive Stereotype (r = 0.984) and with the total score (r = 0.996), indicating possible conceptual overlaps. Physical Attraction and Social Success are strongly related in both groups (GS: r = 0.787; GNS: r = 0.791, *p* < 0.001). Negative Stereotype shows strong correlations with all other dimensions in the non-athletic group (r = 0.702–0.831), but only moderate correlations in the athletic group (r = 0.674–0.879), suggesting that athletes may view negative body stereotypes differently. IBIS-R total scores are strongly linked with subcomponents in the non-athletic group (r = 0.712–0.860) and moderately in the athletic group (r = 0.674–0.765), implying that athletic activity may influence how different parts of ideal body stereotypes relate. These findings indicate that BMI is significantly associated with perceptions of ideal body stereotypes, with subtle differences between athlete and non-athlete groups in the strength and structure of these relationships.

**Table 6:** Correlation Pearson between BMI and Ideal Body Stereotype Scale-Revised (IBIS-R) for GS (the upper right diagonal of the table) and GNS (italic letters—the lower left diagonal of the table).

Variables	Correlation	BMI (kg/m^2^)	Physical Attraction	Social Success	Personal Success	Positive Stereotype	Negative Stereotype	Score Total IBIS-R
BMI (kg/m^2^)	r	-	0.861 **	0.899 **	0.805 **	0.805 **	0.906 **	0.805 **
	*p*	-	0.000	0.000	0.000	0.000	0.000	0.000
Physical Attraction	r	*0.890* **	-	0.787 **	0.716 **	0.716 **	0.803 **	0.716 **
	*p*	*0.000*	-	0.000	0.000	0.000	0.000	0.000
Social Success	r	*0.902* **	*0.791* **	-	0.750 **	0.750 **	0.879 **	0.750 **
	*p*	*0.000*	*0.000*	-	0.000	0.000	0.000	0.000
Personal Success	r	*0.846* **	*0.765* **	*0.860* **	-	0.984 **	0.674 **	0.996 **
	*p*	*0.000*	*0.000*	*0.000*	-	0.000	0.000	0.000
Positive Stereotype	r	*0.846* **	*0.765* **	*0.860* **	*0.723* **	-	0.674 **	0.996 **
	*p*	*0.000*	*0.000*	*0.000*	*0.000*	-	0.000	0.000
Negative Stereotype	r	*0.884* **	*0.795* **	*0.831* **	*0.702* **	*0.706* **	-	0.674 **
	*p*	*0.000*	*0.000*	*0.000*	*0.000*	*0.000*	-	0.000
Score Total IBIS-R	r	*0.846* **	*0.765* **	*0.860* **	*0.721* **	*0.715* **	*0.712* **	-
	*p*	*0.000*	*0.000*	*0.000*	*0.000*	*0.000*	*0.000*	-

** Correlation is significant at the 0.01 level (2-tailed). Italics for GNS; normal letters for GS.

The correlation analysis between BMI and total scores of the main psychometric instruments shows different relationships between the assessed constructs in both study groups. The correlations with BMI reveal distinct patterns of association (Table 7). Body dissatisfaction (SRS-D) exhibits the strongest correlation with BMI in both groups, with slightly higher values in the non-athletic group (GNS: r = 0.940 vs. GS: r = 0.904, *p* < 0.001). Ideal body stereotypes (IBIS-R) are strongly correlated with BMI in the non-athletic group (r = 0.846, *p* < 0.001) compared to the athletic group (r = 0.805, *p* < 0.001). Body objectification (OBC) shows moderate correlations, with a slightly higher magnitude in the non-athletic group (r = 0.394 vs. r = 0.352, *p* < 0.001). Inter-construct relationships indicate significant associations but of different strengths. Body dissatisfaction (SRS-D) is strongly correlated with ideal body stereotypes in both groups (GNS: r = 0.855; GS: r = 0.841, *p* < 0.001), suggesting that body dissatisfaction closely relates to adherence to rigid body ideals. The correlation of SRS-D with body objectification is moderate and similar between groups (GNS: r = 0.333; GS: r = 0.292, *p* < 0.001). Body objectification (OBC) shows the weakest association with ideal body stereotypes, with a significant but weak correlation in the non-athletic group (r = 0.191, *p* = 0.002) and a weak correlation in the athletic group (r = 0.184, *p* = 0.013) (Table 7). This weaker relationship suggests that body objectification and ideal stereotypes are distinct yet interconnected psychological constructs. Differences between groups indicate that non-athletic participants generally show stronger correlations between all assessed constructs, suggesting a closer connection of negative body image experiences. The athletic group exhibits slightly weaker correlations, possibly due to the protective effects of physical activity in moderating the relationships between anthropometric factors and body-related psychological experiences. These findings confirm the link between objective body measurements and subjective body image experiences, with notable differences between athletic and non-athletic populations.

**Table 7:** Correlation Pearson between BMI and Total Score of SRS, OBS and BIS-R for GS and GNS.

Variables	Correlation	BMI (kg/m^2^)	SRS-D	Scor Total OBC	Scor Total IBIS-R
BMI (kg/m^2^)	r	-	0.904 **	0.352 **	0.805 **
	*p*	-	0.000	0.000	0.000
Body Dissatisfaction (SRS-D)	r	*0.940* **	-	0.292 **	0.841 **
	*p*	*0.000*	-	0.000	0.000
Score Total OBC	r	*0.394* **	*0.333* **	-	0.184 *
	*p*	*0.000*	*0.000*	-	0.013
Total Score IBIS-R	r	*0.846* **	*0.855* **	*0.191* **	-
	*p*	*0.000*	*0.000*	*0.002*	-

** Correlation is significant at the 0.01 level (2-tailed). * Correlation is significant at the 0.05 level (2-tailed). Italics for GNS; normal letters for GS.

In Table 8, the comparative analysis of linear regression models between the sports (GS) and non-sports (GNS) groups reveals consistent differences in the predictive abilities and the strength of the relationships between the studied variables. The linear regression analysis shown in Table 8 illustrates varying predictive abilities of the studied variables on psychological constructs related to body image in both groups. Analysis of the coefficients of determination (R^2^) reveals substantial variability in the predictive power of the independent variables. Current Size (SRS-C) demonstrates the strongest predictive capacity, explaining 87.8% (GS) and 91.6% (GNS) of the BMI variance, and 80.1% (GS) and 86.3% (GNS) of the Body Dissatisfaction variance, respectively (F = 717.617–2778.935, *p* < 0.001). Body Surveillance and Body Shame present R^2^ coefficients higher than 0.80 for BMI and SRS-D in both groups, confirming robust predictive relationships.

**Table 8:** Linear regression of the variables of the study.

			Group Sportive (GS)				Group Non-Sportive (GNS)			
Predictor	Statistics		BMI	SRS-D	Total Score OBC	Total Score IBIS-R	BMI	SRS-D	Total Score OBC	Total Score IBIS-R
Current Size (SRS-C)	Regression	R^2^	0.878	0.801	0.076	0.702	0.916	0.863	0.089	0.754
	Anova	F	1285.888	717.617	14.744	419.006	2778.935	1605.014	24.748	778.134
		*p*	0.000 ^b^	0.000 ^b^	0.000 ^b^	0.000 ^b^	0.000 ^b^	0.000 ^b^	0.000 ^b^	0.000 ^b^
	β	β	0.937	0.895	0.277	0.838	0.957	0.929	0.298	0.868
Ideal Size (SRS-I)	Regression	R^2^	0.080	0.000 ^b^	0.001	0.036	0.088	0.006	0.002	0.069
	Anova	F	15.456	0.005	0.192	6.466	24.459	1.423	0.010	18.713
		*p*	0.000 ^b^	0.944 ^b^	0.661 ^b^	0.014 ^b^	0.000 ^b^	0.234 ^b^	0.919 ^b^	0.000 ^b^
		β	0.283	0.151	0.033	0.189	0.296	0.075	0.068	0.262
Body Dissatisfaction (SRS-D)	Regression	R^2^	0.817	-	0.851	0.708	0.883	-	0.711	0.732
	Anova	F	794.823	-	16.573	431.402	1925.786	-	31.710	692.955
		*p*	0.000 ^b^	-	0.000 ^b^	0.000 ^b^	0.000 ^b^	-	0.000 ^b^	0.000 ^b^
		β	0.904	-	0.292	0.839	0.940	-	0.332	0.855
Body surveillance	Regression	R^2^	0.873	0.810	0.084	0.539	0.889	0.869	0.086	0.647
	Anova	F	1225.263	759.399	16.252	208.450	2043.815	1683.933	23.977	464.960
		*p*	0.000 ^b^	0.000 ^b^	0.000 ^b^	0.000 ^b^	0.000 ^b^	0.000 ^b^	0.000 ^b^	0.000 ^b^
		β	0.934	0.900	0.289	0.737	0.943	0.932	0.294	0.804
Body shame	Regression	R^2^	0.844	0.780	0.080	0.534	0.841	0.808	0.090	0.628
	Anova	F	968.246	632.619	15.511	195.683	1346.641	1066.120	24.988	428.173
		*p*	0.000 ^b^	0.000 ^b^	0.000 ^b^	0.000 ^b^	0.000 ^b^	0.000 ^b^	0.000 ^b^	0.000 ^b^
		β	0.919	0.883	0.283	0.726	0.917	0.899	0.299	0.792
Appearance body control	Regression	R^2^	0.853	0.787	0.050	0.526	0.840	0.834	0.042	0.629
	Anova	F	1032.607	658.190	9.302	197.727	1337.889	1271.831	11.179	431.044
		*p*	0.000 ^b^	0.000 ^b^	0.003 ^b^	0.000 ^b^	0.000 ^b^	0.000 ^b^	0.000 ^b^	0.000 ^b^
		β	0.924	0.887	0.233	0.727	0.916	0.913	0.205	0.793
Totale Score OBC	Regression	R^2^	0.124	0.0850	-	0.034	0.156	0.111	-	0.036
	Anova	F	25.243	16.573	-	6.254	46.770	31.710	-	9.610
		*p*	0.000 ^b^	0.000 ^b^	-	0.013 ^b^	0.000 ^b^	0.000 ^b^	-	0.002 ^b^
		β	0.356	0.292	-	0.184	0.394	0.333	-	0.191
Physical Attraction	Regression	R^2^	0.741	0.577	0.078	0.513	0.793	0.720	0.102	0.585
	Anova	F	510.116	242.468	15.113	187.137	971.007	651.634	28.880	357.840
		*p*	0.000 ^b^	0.000 ^b^	0.000 ^b^	0.000 ^b^	0.000 ^b^	0.000 ^b^	0.000 ^b^	0.000 ^b^
		β	0.830	0.728	0.269	0.708	0.890	0.848	0.320	0.765
Social Success	Regression	R^2^	0.808	0.783	0.054	0.563	0.814	0.791	0.045	0.739
	Anova	F	748.108	640.402	10.129	229.216	1114.623	964.199	11.957	718.439
		*p*	0.000 ^b^	0.000 ^b^	0.000 ^b^	0.000 ^b^	0.000 ^b^	0.000 ^b^	0.000 ^b^	0.000 ^b^
		β	0.899	0.885	0.232	0.752	0.902	0.890	0.212	0.860
Personal Success	Regression	R^2^	0.649	0.708	0.034	0.898	0.715	0.732	0.036	0.923
	Anova	F	328.811	431.402	6.254	798.168	1532.983	692.955	9.610	836.453
		*p*	0.000 ^b^	0.000 ^b^	0.013 ^b^	0.000 ^b^	0.000 ^b^	0.000 ^b^	0.000 ^b^	0.000 ^b^
		β	0.796	0.827	0.182	0.994	0.846	0.855	0.191	0.984
Positive Stereotype	Regression	R^2^	0.6519	0.711	0.037	0.867	0.714	0.733	0.038	0.917
	Anova	F	3318.817	437.478	6.412	771.572	638.472	695.124	9.694	831.216
		*p*	0.000 ^b^	0.000 ^b^	0.016 ^b^	0.000 ^b^	0.000 ^b^	0.000 ^b^	0.000 ^b^	0.000 ^b^
		β	0.806	0.839	0.184	0.985	0.826	0.850	0.199	0.965
Negative Stereotype	Regression	R^2^	0.822	0.697	0.070	0.454	0.781	0.747	0.081	0.489
	Anova	F	820.536	409.472	13.362	147.888	906.503	750.525	22.300	252.144
		*p*	0.000 ^b^	0.000 ^b^	0.000 ^b^	0.000 ^b^	0.000 ^b^	0.000 ^b^	0.000 ^b^	0.000 ^b^
		β	0.906	0.835	0.264	0.675	0.884	0.864	0.284	0.706
Score Total IBIS-R	Regression	R^2^	0.649	0.708	0.034	-	0.715	0.731	0.036	
	Anova	F	328.811	431.402	6.254	-	638.472	692.955	9.610	-
		*p*	0.000 ^b^	0.000 ^b^	0.013 ^b^	-	0.000 ^b^	0.000 ^b^	0.002 ^b^	-
		β	0.806	0.839	0.184	-	0.864	0.865	0.192	-

R^2^—R Square, *p*—level of probability, β—standardized beta coefficients; ^b^ Correlation is significant at the 0.05 level (2-tailed).

Body Dissatisfaction functions as a strong predictor for Total Score OBC (R^2^ = 0.851, GS; R^2^ = 0.711, GNS) and IBIS-R (R^2^ = 0.708, GS; R^2^ = 0.732, GNS), all models being statistically significant (*p* < 0.001). The IBIS-R dimensions (Physical Attraction, Social Success, Personal Success, Positive/Negative Stereotypes) show high predictive power for total IBIS-R (R^2^ > 0.65), with Personal Success and Positive Stereotype reaching exceptional values (R^2^ > 0.89 for both groups). Contrary to expectations, Ideal Size (SRS-I) shows minimal predictive capacity (R^2^ < 0.09 for most variables), with non-significant models for SRS-D in GS (*p* = 0.944) and GNS (*p* = 0.234), and for OBC in GS (*p* = 0.661). The Total OBC Score as a predictor explains only 3.4–15.6% of the variance in the other variables, although the models remain statistically significant.

Current Size → BMI exemplifies this pattern (R^2^ = 0.916, GNS vs. R^2^ = 0.878, GS). All F-values are significant (*p* < 0.001), confirming the statistical validity of the regression models for both groups. The results highlight the predominance of current body perception over ideal in predicting psychometric and anthropometric variables, with consistent but differentiated patterns between athletic and non-athletic individuals. The non-athletic group consistently displays higher F values, indicating stronger and more stable relationships among variables. This difference suggests that in the sedentary population, anthropometric and psychological factors are more closely connected, possibly due to the absence of modulatory effects of physical activity.

The analysis of standardized beta coefficients reveals substantial differences in the magnitude of associations between predictors and dependent variables. Body Dissatisfaction (SRS-D) presents the strongest associations, with β values > 0.88 for most relationships in both groups, indicating a very strong link with BMI (β = 0.904 GS; β = 0.940 GNS), Body Surveillance (β = 0.900 GS; β = 0.932 GNS), and Body Appearance Stereotypes (β = 0.839 GS; β = 0.855 GNS). The Body Surveillance and Body Shame dimensions show similar patterns of very strong associations (β > 0.80) with all variables analyzed. In contrast, Ideal Size (SRS-I) registers the lowest beta coefficients (β = 0.033–0.296), suggesting a limited impact on the other investigated dimensions. Total OBC Score shows moderate associations (β = 0.205–0.394), with the weakest relationships identified for Total IBIS-R Score (β = 0.184 GS; β = 0.191 GNS). The inter-group comparison reveals slightly higher β values in the non-athletic group for most relationships (differences of 0.01–0.05), indicating marginally stronger associations between psychological body image variables, although the overall patterns remain consistent in both samples. These results show that negative body image dimensions (dissatisfaction, surveillance, shame) are strongly associated with anthropometric and cognitive indicators of body perception, while behavioral control and perceived ideal size exert more moderate influences.

## 4. Discussion

The aim of the study was to assess multidimensional differences in body image between female students who regularly practice sports and those who are sedentary. This was performed by analyzing the correlations between objective measures like BMI and subjective body image perceptions, with the goal of identifying protective factors and establishing theoretical bases for intervention programs. The study’s findings on the multidimensional relationship between body image and activity levels in both athletic and non-athletic female students support and expand existing scientific evidence concerning the link between physical activity and body perceptions within the specific context of Romanian university students. Our results support the strong body of research showing the positive effects of physical exercise on body image (Magel et al., 2025; Gualdi-Russo et al., 2024). Recent studies also identify a significant positive correlation between physical activity and body image in university students, mediated by factors such as self-esteem and social adjustment (Gualdi-Russo et al., 2022b; Goicochea et al., 2022). In-depth research on Chinese university students indicates that physical activity influences body image both directly and indirectly through the development of athletic body models, which aligns with our findings (Wang et al., 2023).

Our results show that 83.9% of participants in the athletic group have a normal BMI compared to 58.2% in the non-athletic group. These findings support recent research indicating that participation in sports during university can help protect against body image concerns (Wang et al., 2023; Holland & Tiggemann, 2016). A recent meta-analysis found that athletes generally report fewer body image concerns than non-athletes, with significant differences based on gender and smaller differences based on weight status (Zaccagni & Gualdi-Russo, 2023). The differences in body satisfaction, with 31.7% of athletic participants expressing perfect satisfaction compared to none in the non-athletic group, further support the idea that motivation to exercise strongly influences the connection between physical activity frequency and a positive body image, as shown in various studies on female students (Hardie et al., 2022; Tylka & Wood-Barcalow, 2015; Wood-Barcalow et al., 2010). A study on Palestinian female students confirmed that those who exercised regularly had significantly higher body appreciation scores than those who did not (Abu Alwafa & Badrasawi, 2023). Similar low scores on the OBC scale across groups suggest that body objectification is influenced more by social factors than physical activity alone, aligning with objectification theory, which explains that social objectification leads to self-objectification by adopting an external perspective on one’s body (Saunders et al., 2024; Lacroix et al., 2023). Additionally, a study involving 594 women highlighted the combined positive effects of physical activity and healthy eating habits in improving body image and reducing the negative impact of social media-induced self-objectification on body image and mental health (Çınaroğlu & Yılmazer, 2025). These findings support our results, which indicate weaker correlations between body objectification and other constructs among athletes.

The strong psychometric properties of the instruments used are consistent with the recent validation of the Silhouette Rating Scale (SRS) as a reliable tool for assessing body size perception and body dissatisfaction, as shown in previous studies (Fernandes et al., 2023; Lombardo et al., 2022). In our study of The Objectified Body Consciousness Scale (OBC) (n.d.), we found excellent internal consistency, similar to earlier validations of this instrument. McKinley and Hyde (1996) developed this scale to measure how women perceive their bodies as objects that can be altered after internalizing social expectations, through three subcomponents: body surveillance, body shame, and control beliefs over physical appearance. The IBIS-R scale demonstrated very good reliability, confirming its usefulness in measuring ideal body stereotypes.

Several studies have examined the links between body image and perceived physical fitness, revealing notable gender differences in body perceptions (Tsartsapakis et al., 2024; Martin & Racine, 2017). Recent research consistently shows that women experience greater body dissatisfaction than men, regardless of division level and sport type (He et al., 2020; Milano et al., 2020; Rabito Alcón & Rodríguez Molina, 2015). A recent factor analysis of college athletes found that women report more body dissatisfaction than men, regardless of division level and sport type, with an interaction between gender and sport type indicating that men in weight-loss sports report greater dissatisfaction than men in non-weight-loss sports (Milligan & Pritchard, 2006). In our study, we aimed to identify relevant aspects of body image among female athletes and non-athletes, but an intergender analysis would be a valuable direction for future research. The observed differences suggest that participation in sports may offer protective effects, reducing the strength of negative links between body traits and adverse psychological experiences.

### 4.1. Study Limitations

The cross-sectional design of the study limits causal inferences between the investigated variables, and future research would benefit from using prospective designs to examine the directionality of relationships, especially to understand how sports activity influences the development of body image over time. The relatively large sample size and the inclusion of only female subjects meant that male subjects were not included for cross-gender analysis. Recent criticisms of BMI as a single measure of anthropometric assessment (World Health Organization, 2021) suggest the need to include additional measurements such as waist circumference or body composition for a more comprehensive evaluation. The lack of longitudinal data limits understanding of the stability of the identified body image relationships over time, and the complexity of the topic requires assessing medium- and long-term changes in body perceptions in both sports and non-sports settings. Focusing solely on female participants reduces the generalizability of the results. Recent studies highlight significant differences between male and female student-athletes’ perceptions of weight and body image, emphasizing the need for detailed comparative analyses. Self-report scales may be influenced by social desirability bias, especially in academic and age-specific contexts where participants might feel pressure to conform to body ideals. Using Google Forms for psychometric data collection may entail uncontrolled administration conditions, including variability in screen size, privacy, and potential environmental distractions. Such factors may affect the reliability of responses, particularly considering the sensitive nature of body image assessments. The lack of control for potential confounding variables (e.g., socioeconomic status, media exposure, dietary habits), which may influence both BMI and body image outcomes, could be considered a limitation.

### 4.2. Practical Implications of the Study and Future Research Directions

The practical implications highlight the importance of developing integrated programs that promote both physical health and psychological well-being among young adults. The high correlations observed between BMI and body perceptions may reflect shared method variance or measurement overlap, considering the complex interaction between anthropometric, psychological, and socio-cultural factors.

Psychological education and body acceptance interventions are essential for both athletes and non-athletes and need to be customized according to their specific characteristics. In athletes, they prevent overinvestment in physical appearance and performance-related stress, and in non-athletes, they reduce vulnerability to depression, eating disorders, and compensatory behaviors, thus strengthening a healthy relationship with one’s own body.

Future studies should use prospective longitudinal designs to explore how body image varies by gender in relation to different physical and sports activities, across different age groups, and in diverse socio-demographic contexts. Experimental research on specific interventions may help clarify the causal mechanisms by which sports activity influences body image, especially given that recent studies show that body surveillance and body shame contribute to the prediction of eating disorders independently of other socio-cultural risk factors.

## 5. Conclusions

The results confirm the statistical associations of sports activity, highlighting weaker correlations between negative constructs in the sports group (for body objectification) and a more favorable distribution of body satisfaction. Our findings offer valuable insight into how sports activity can act as a protective factor against the development of negative body image concerns. Results suggest that participating in sports is linked to a healthier anthropometric profile and a more positive body image, characterized by increased satisfaction and more realistic perceptions of body size. The analysis of the groups included in the study (GS vs. GNS) reveals significant differences in body dissatisfaction, body shame, social success, body control, body size perception, and body surveillance. The study identified moderate differences between the two groups in negative stereotypes, personal success, positive stereotypes, IBIS-R total score, and physical attractiveness. The study recorded minimal differences between the groups regarding ideal body image and total OBC score. Psychometric profiles highlight the statistical associations consistent with protective patterns of sports activity, with measurable impacts: athletes have anthropometric traits within the healthy BMI range with moderate improvements, realistic body perceptions with significantly increased satisfaction, notably lower levels of objectification, improved body control, and substantially decreased adherence to body stereotypes. The results of the study suggest that in both groups, there is a strong agreement between objective measurements (BMI) and subjective perceptions of body size for the SRS (Silhouette Rating Scale). However, there are subtle differences in how body ideals influence body satisfaction between athletic and non-athletic populations. The correlation analysis between BMI and the OBC scale (Objectified Body Consciousness) reveals complex patterns of association that vary significantly between sports and non-sports groups. The results confirm that body objectification is closely related to anthropometric characteristics, but with slightly different magnitudes that may reflect the protective effects of sports activity. The correlation analysis between BMI and the IBIS-R (Ideal Body Stereotype Scale-Revised) scale shows strong and consistent associations between anthropometric characteristics and ideal body stereotypes in both study groups. The correlations with BMI demonstrate strong and positive relationships with all dimensions of body stereotypes in the sports group (GS). Body objectification (OBC) shows the weakest association with ideal body stereotypes, with a significant but weak correlation in the non-sports group and a weak correlation in the sports group. This weaker relationship suggests that body objectification and ideal stereotypes are distinct, yet interconnected, psychological constructs. The differences between groups indicate that non-athletic participants generally exhibit stronger correlations across all assessed constructs, suggesting a closer connection of negative experiences related to body image.

Comparative analysis of linear regression models between the sports group (GS) and the non-sports group (GNS) reveals consistent differences in terms of the strength of associations between the studied variables. The results for the non-athletic group (sedentary and semi-sedentary) show stronger connections between the variables. This difference suggests that, in the sedentary population, anthropometric and psychological factors are more closely related, possibly due to the absence of modulatory effects of irregular and insufficient physical activity (physically inactive subjects or those involved in less than 2 h of physical activities per week with low intensity and volume of effort). Athletes tend to base their body image on functionality and performance, which favors a more realistic and healthy evaluation, while non-athletes are more exposed to aesthetic standards and social pressures. These results highlight the multifactorial nature of the relationships between anthropometric parameters, body cognitions, and psychological dimensions of body image, with regression models demonstrating variable predictive capacities for the investigated factors.

## Figures and Tables

**Table 1:** Distribution of study samples in BMI and with the questionnaires applied in the study.

Parameters/ Questionnaire	Score Interpretation	Classification	Group Sportive (GS)		Group Nonsportive (GNS)	
			No.	Percent	No.	Percent
BMI * (kg/m^2^)	<18.5	Underweight	-	-	-	-
	18.5–24.99	Normoweight	151	83.9%	149	58.2%
	25–29.99	Overweight	29	16.1%	106	41.4%
	30–34.99	Grade 1 obesity	-	-	1	0.4%
	35–39.99	Grade 2 obesity	-	-	-	-
	>40	Grade 3 obesity	-	-	-	-
Silhouette Rating Scale (SRS)	SRS-C (Scale 1–9):					
	1–3	Thin body size perception	42	23.3%	28	10.9%
	4–6	Average body size perception	119	66.1%	155	60.6%
	7–9	Large body size perception	19	10.6%	73	28.5%
	Body dissatisfaction (SRS-D):					
	0	Perfect body satisfaction	57	31.7%	-	-
	±1–2	Mild body dissatisfaction	92	51.1%	8	3.1%
	±3–4	Moderate body dissatisfaction	29	16.1%	248	96.9%
	±5–8	Severe body dissatisfaction	2	1.1%	-	-
Objectified Body Consciousness Scale (OBC)	OBC (Scale 1–7):					
	1–2	Very low level of body objectification	-	-		
	3–4	Low level of body objectification	178	98.9%	248	96.9%
	5	Moderate level of body objectification	2	1.1%	8	3.1%
	6	Moderate-high level of body objectification	-	-		
	7	High level of body objectification	-	-		
Ideal Body Stereotype Scale-Revised (IBIS-R)	Scale IBIS-R (Scale 1–5):					
	1	Very low	-	-	-	-
	2	Low-moderate	57	31.7%	37	14.5%
	3	Moderate	95	52.8%	115	44.9%
	4	Moderate-high	28	15.6%	104	40.6%
	5	High	-	-		

* Body mass index (BMI) classification, according to WHO (World Health Organization) standards.

**Table 2:** Descriptive Statistics of the variable of the study.

Categories	Variables	Groups	Min.	Max.	Mean	SD	Skewness	Kurtosis	Shapiro–Wilk-*p*	α
BMI	BMI (kg/m^2^)	GS	19.80	31.20	22.285	2.251	0.513	0.734	0.811 *	-
		GNS	19.80	31.20	24.159	2.716	0.312	−0.602	0.957 *	-
Silhouette Rating Scale (SRS)	Current Size (SRS-C)	GS	3.00	8.00	4.538	1.330	0.866	0.197	0.876 *	0.871
		GNS	3.00	8.00	5.543	1.483	−0.073	−0.895	0.933 *	0.791
	Ideal Size (SRS-I)	GS	2.00	4.00	3.316	0.593	−0.231	−0.617	0.747 *	0.802
		GNS	2.00	4.00	3.394	0.550	−0.143	−0.918	0.710 *	0.819
	Body Dissatisfaction (SRS-D)	GS	0.00	5.00	1.222	1.193	0.956	0.305	0.845 *	0.784
		GNS	0.00	5.00	2.144	1.337	−0.017	−0.720	0.922 *	0.769
Objectified Body Consciousness Scale (OBC)	Body surveillance	GS	3.00	6.00	3.555	0.898	0.328	0.398	0.646 *	0.876
		GNS	3.00	6.00	4.335	1.019	−0.108	−1.275	0.832 *	0.952
	Body shame	GS	2.00	6.00	2.650	1.070	0.291	0.164	0.634 *	0.876
		GNS	2.00	6.00	3.621	1.204	−0.253	−1.259	0.819 *	0.879
	Appearance body control	GS	2.00	6.00	5.216	1.295	−0.252	−0.113	0.623 *	0.793
		GNS	2.00	6.00	4.027	1.448	0.389	−1.440	0.791 *	0.807
	Score Total OBC	GS	4.00	5.00	4.011	0.105	0.407	0.455	0.080 *	0.968
		GNS	4.00	5.00	4.031	0.174	1.420	1.592	0.164 *	0.813
Ideal Body Stereotype Scale-Revised (IBIS-R)	Physical Attraction	GS	1.00	5.00	3.222	0.575	0.766	1.792	0.662 *	0.874
		GNS	2.00	5.00	3.550	0.605	0.071	−0.376	0.770 *	0.824
	Social Success	GS	2.00	4.00	2.433	0.717	0.337	0.261	0.618 *	0.861
		GNS	2.00	4.00	3.039	0.815	−0.072	−1.493	0.793 *	0.819
	Personal Success	GS	2.00	4.00	2.838	0.669	0.199	−0.779	0.793 *	0.827
		GNS	2.00	4.00	3.261	0.695	−0.405	−0.891	0.785 *	0.811
	Positive Stereotype	GS	1.00	4.00	2.886	0.696	0.199	−0.779	0.793 *	0.789
		GNS	2.00	4.00	3.261	0.695	−0.405	−0.891	0.785 *	0.812
	Negative Stereotype	GS	2.00	4.00	2.272	0.493	0.581	0.599	0.567 *	0.836
		GNS	2.00	4.00	2.722	0.571	0.080	−0.507	0.736 *	0.892
	Score Total IBIS-R	GS	2.00	4.00	2.890	0.688	0.197	−0.773	0.791 *	0.931
		GNS	2.00	4.00	3.261	0.695	−0.405	−0.891	0.785 *	0.872

GS—group sportive, GNS—group non-sportive, Min—minimum, Max—Maximum, SD—standard deviation, BMI—body mass index; α—Reliability analysis, *—statistically significant at *p* < 0.05.

## Data Availability

Data are contained within the article.
